# The Gut Microbiome and Colorectal Cancer: From Association to Causation

**DOI:** 10.66505/cbtt.v1i2.40

**Published:** 2026-05-12

**Authors:** Toshiaki Takahashi, Ajay Goel

**Affiliations:** 1. Department of Molecular Diagnostics and Experimental Therapeutics, Beckman Research Institute of City of Hope, Biomedical Research Center, Monrovia, California, USA; 2. City of Hope Comprehensive Cancer Center, Duarte, California, USA

**Keywords:** colorectal cancer, gut microbiome, Fusobacterium nucleatum, tumor microenvironment, immunotherapy sensitivity, microbiome-based biomarkers

## Abstract

This mini-review discusses the emerging role of the gut microbiome as an active driver of colorectal cancer initiation, progression, and therapeutic response. Key mechanisms include microbiome-induced genomic instability, modulation of host immune responses, and epigenetic reprogramming mediated by tumor-associated bacteria such as *Fusobacterium nucleatum*. Emerging evidence suggests that specific microbial signatures are not only associated with disease but can functionally shape tumor behavior, influence treatment sensitivity, and serve as clinically actionable biomarkers. These insights highlight the potential of integrating microbiome profiling into precision oncology and underscore the need for mechanistic and translational studies to harness host–microbe interactions for improved cancer prevention and therapy.

## 1. Introduction

**Figure 1.**
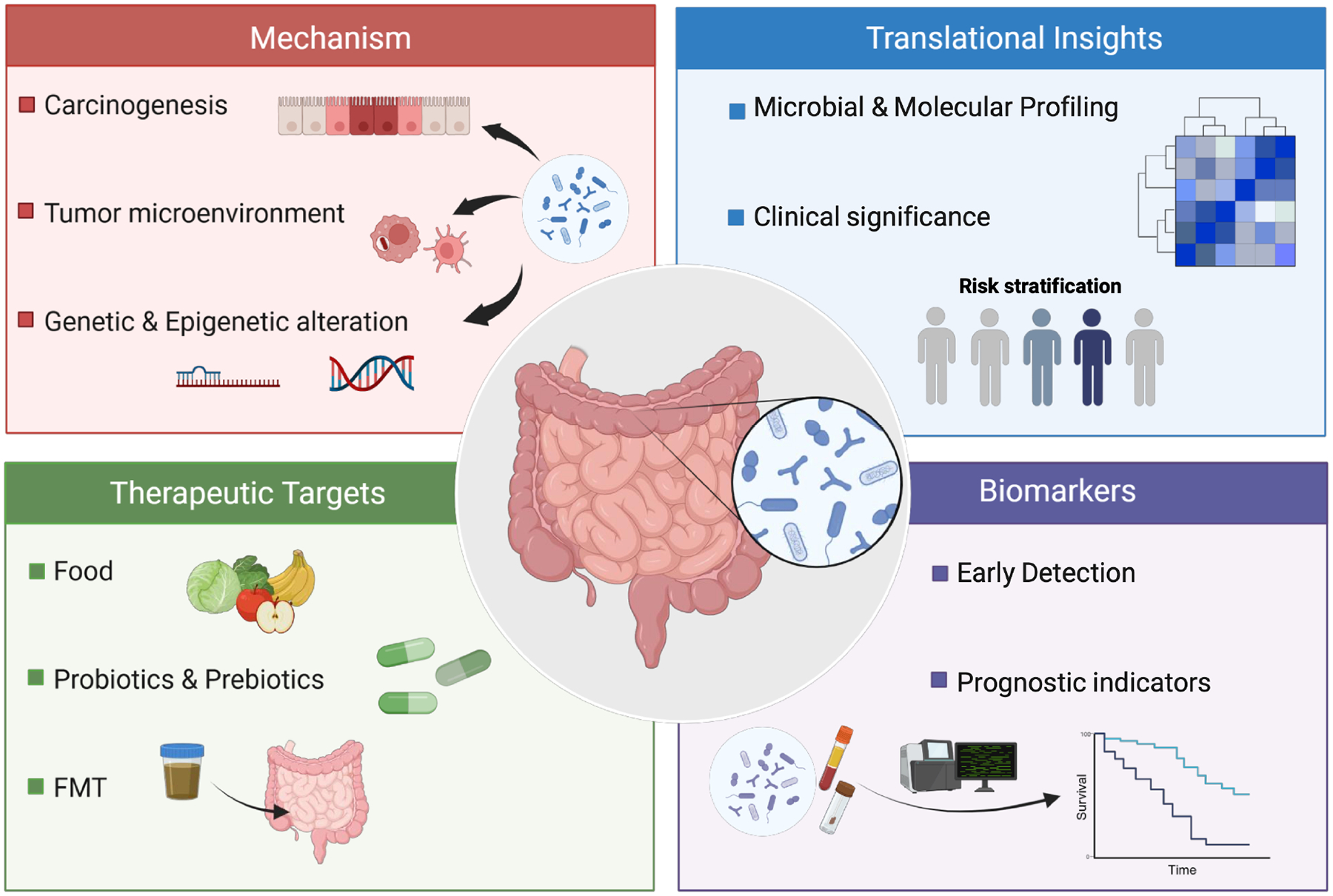
Framework linking the gut microbiome to cancer biology and clinical translation. The schematic depicts the gastrointestinal tract and associated microbial communities as a source of host-microbe interactions that influence cancer risk and outcomes. Key mechanisms include microbiome-associated processes that can promote carcinogenesis, shape the tumor microenvironment, and contribute to genetic and epigenetic alterations through microbe-derived signals and host inflammatory responses. Translational insights arise from integration of microbial and molecular profiling (for example, sequencing-based community signatures and host pathway readouts) with clinical interpretation, including patient risk stratification. The framework highlights therapeutic targets representing modifiable intervention points to alter microbiome composition or function, including diet/food-based approaches, probiotics and prebiotics, and fecal microbiota transplantation. The schematic also illustrates microbiome-informed biomarkers to support early detection and prognostic assessment, including biospecimen testing workflows and survival/outcome analyses. Abbreviations: FMT, fecal microbiota transplantation.

Colorectal cancer (CRC) remains one of the leading causes of cancer morbidity and mortality worldwide, despite advances in screening, surgical techniques, and systemic therapies. Traditionally, colorectal carcinogenesis has been understood through the lens of host genetics, somatic mutations, diet, and chronic inflammation. However, over the past decade, a paradigm shift has occurred. The gut microbiome, once considered a passive inhabitant of the intestinal lumen, has emerged as an active biological participant in colorectal tumor initiation, progression, and therapeutic response (1–3). This evolving understanding challenges long-held assumptions about cancer biology and compels a more integrated view of host-microbe interactions. Increasing evidence now supports the concept that specific microbial communities, in addition to generalized dysbiosis alone, can reprogram the tumor microenvironment, modulate immune surveillance, and influence molecular signaling pathways fundamental to colorectal carcinogenesis (4). Accumulating evidence on host-microbiome interactions suggests that microbiome-based molecular profiling may serve as a non-invasive tool for diagnosis, prognosis, and therapeutic decision-making in CRC, as illustrated in Figure 1. Against this conceptual backdrop, the following section examines how disruption of the normal gut microbial ecosystem—dysbiosis—contributes directly to colorectal tumor initiation and progression.

### 1.1 Microbiome Dysbiosis in Colorectal Carcinogenesis

The healthy colon harbors a complex and diverse microbial ecosystem that supports epithelial integrity, immune homeostasis, and metabolic balance. In CRC, this equilibrium is disrupted. Tumor-associated microbiomes are consistently characterized by reduced microbial diversity and selective enrichment of pro-inflammatory and genotoxic organisms (5). These alterations are not merely consequences of malignancy; longitudinal and mechanistic studies increasingly indicate that microbial shifts may precede and promote neoplastic transformation (6). Several bacterial taxa have been implicated in CRC, including enterotoxigenic *Bacteroides fragilis* (ETBF), colibactin-producing Escherichia coli (E. coli), and, most prominently, *Fusobacterium nucleatum* (*F. nucleatum*). E. coli strains harboring the pks island produce colibactin, a genotoxin that induces DNA damage and characteristic mutational signatures in CRC (7). Enterotoxigenic *Bacteroides fragilis*, which produces the Bacteroides fragilis toxin (BFT), also contributes to tumorigenesis via inflammation-mediated pathways (8). Among these, *F. nucleatum* has the most consistent association with colorectal tumors across geographic regions, disease stages, and molecular subtypes, positioning it as a central figure in microbiome-driven CRC oncogenesis (4, 9–13).

### 1.2 Fusobacterium nucleatum as an Active Driver of Colorectal Carcinogenesis

*F. nucleatum* is an anaerobic, Gram-negative bacterium commonly found in the oral cavity and frequently enriched in colorectal adenomas and carcinomas (11). Its presence in tumor tissue correlates with advanced disease stage, higher recurrence rates, and poorer overall survival, suggesting biological relevance beyond opportunistic colonization (12). Recent studies indicate the existence of a CRC-enriched clade of F. nucleatum, suggesting its involvement in tumorigenesis (14). Mechanistic studies have elucidated several pathways by which *F. nucleatum* promotes tumor progression (9). *F. nucleatum* adheres to E-cadherin on host epithelial cells and promotes CRC development via the fusobacterium adhesion factor FadA (15). Recently, *F. nucleatum* has been shown to disrupt epithelial cell-cell interactions and induce cell-cycle arrest (16). Additionally, the bacterium engages pattern recognition receptors on colonic epithelial cells, activating inflammatory cascades such as NF-κB signaling (4). This sustained inflammatory milieu fosters genomic instability, enhances cellular proliferation, and facilitates tumor progression.

Concurrently, *F. nucleatum* exerts potent immunomodulatory effects by regulating the tumor microenvironment. Secreted signaling molecules interact with macrophages and cytotoxic T lymphocytes, attenuating antitumor immune responses and enabling immune evasion (4, 17, 18). This immunosuppression is particularly significant in the context of emerging immunotherapy strategies, as microbiota composition may partially explain variability in response to immune checkpoint inhibitors in CRC. Furthermore, by acting through cell-surface receptors, *F. nucleatum* induces autophagy and suppresses cell death, thereby promoting cancer. Although *F. nucleatum* has been widely characterized as immunosuppressive, recent evidence paradoxically suggests that it may facilitate response to immune checkpoint blockade in microsatellite-stable CRC (19).

This microbial-epigenetic crosstalk provides a direct molecular link between bacterial presence and malignant cellular behavior, reinforcing the concept of bacteria as functional components of the tumor microenvironment (20).

### 1.3 Translational Insights from Host-Microbiome Molecular Integration

The integration of microbiome science with molecular oncology has accelerated the transition from descriptive associations to actionable insights. Translational studies combining microbial profiling with host genomic and epigenomic data have shown that *F. nucleatum*-associated tumors exhibit distinct molecular features, including altered microRNA expression patterns, inflammatory gene signatures, and resistance to certain chemotherapeutic agents (10, 21). One particularly impactful line of research has demonstrated a functional synergy between *F. nucleatum* abundance and oncogenic microRNA signaling. Elevated levels of both the bacterium and miR-21 have been associated with accelerated tumor growth and poorer clinical outcomes, whereas experimental suppression of miR-21 mitigates the bacterium's tumor-promoting effects (10). Beyond inflammation and immunoregulation, *F. nucleatum* also influences host gene expression. In experimental models, bacterial colonization has been shown to suppress tumor-suppressing pathways while upregulating oncogenic microRNAs, particularly miR-21 (10). Furthermore, it has been suggested that *F. nucleatum* may promote carcinogenesis by influencing DNA methylation within CpG islands (22). Recent studies on inflammation-associated tumorigenesis suggest that macrophage-mediated RNA editing contributes to oncogenic transcriptome remodeling (20, 23, 24).

In this context, *F. nucleatum* is thought to promote CRC development through mechanisms that may involve RNA editing. These findings underscore the importance of viewing CRC as a disease shaped by both microbial and host molecular determinants. Through crosstalk with the host, the microbial-epigenetic interactions demonstrated by *F. nucleatum* reveal a direct molecular-level link between bacterial presence and malignant cellular behavior, reinforcing the concept that bacteria are functional components of the tumor microenvironment.

Such integrative approaches have opened new avenues for biomarker development. Microbial signatures detectable in stool, tissue, or circulating nucleic acids may complement existing screening modalities and improve early detection, especially in patients with molecularly aggressive disease. Moreover, combined microbial-molecular profiles may enable more accurate prognostication and patient stratification (25).

### 1.4 Clinical Implications and Therapeutic Opportunities

Building on these molecularly informed insights, attention has increasingly shifted toward leveraging microbiome knowledge in clinical practice. Recognition of the microbiome’s role in CRC carries significant clinical implications. First, microbiome-based biomarkers have the potential to enhance non-invasive screening strategies and identify high-risk individuals before malignant transformation (25). Second, microbial composition may serve as a predictive factor for therapeutic response, influencing sensitivity to chemotherapy, radiotherapy, and immunotherapy (26).

Perhaps most compelling is the prospect of microbiome-targeted interventions. Dietary modulation, prebiotics, probiotics, selective antibiotics, and fecal microbiota transplantation are all under investigation as strategies to restore microbial balance and suppress tumor-promoting organisms. While these approaches remain experimental, they represent a shift toward biologically informed, preventive oncology. However, caution is warranted. The microbiome is highly individualized and context-dependent, shaped by diet, genetics, environment, and medication exposure. Furthermore, identifying truly influential biomarkers remains challenging, as it requires consideration of confounding factors unique to microbiome research (27). Broad, nonspecific interventions may yield unpredictable effects, underscoring the need for precision strategies guided by robust mechanistic understanding.

## Discussion

Despite rapid progress, several challenges remain. First, establishing causality in human populations is inherently complex, and disentangling cause from consequence requires carefully designed longitudinal studies. Second, accurate biomarker development requires quantitative microbiome profiling with appropriate covariate control, and standardizing microbiome sampling, sequencing, and analytical pipelines is essential to ensure reproducibility and clinical applicability (27). Moreover, future research must move beyond cataloging microbial taxa to functional characterization of microbial metabolites, virulence factors, and host signaling interactions. Ultimately, integrating microbiomics with transcriptomics, metabolomics, and immune profiling will be critical to fully elucidate the microbiome’s role in CRC biology.

In conclusion, the gut microbiome has emerged as a critical, previously underappreciated determinant of CRC development and progression. *F. nucleatum* exemplifies how specific microbes can actively shape tumor biology through inflammatory, immune, and molecular mechanisms. As translational research continues to integrate microbial and host data, the microbiome is poised to become a central pillar of CRC prevention, diagnosis, and personalized therapy. Embracing this complexity is not only a scientific challenge but also a profound opportunity to redefine how CRC is understood and treated.

## Data Availability

“No datasets were generated or analyzed in the current study.”
